# Alpha-Synuclein and Its Role in Melanocytes

**DOI:** 10.3390/cells11132087

**Published:** 2022-06-30

**Authors:** Nicole Rachinger, Nora Mittag, Ines Böhme-Schäfer, Wei Xiang, Silke Kuphal, Anja K. Bosserhoff

**Affiliations:** 1 Institute of Biochemistry, Friedrich-Alexander-Universität Erlangen-Nürnberg (FAU), 91054 Erlangen, Germany; nicole.rachinger@fau.de (N.R.); ines.boehme@fau.de (I.B.-S.); silke.kuphal@fau.de (S.K.); 2 Department of Dermatology and Allergy, University Hospital, LMU Munich, 80539 Munich, Germany; nora.mittag@med.uni-muenchen.de; 3 Department of Molecular Neurology, University Hospital Erlangen, Friedrich-Alexander-Universität Erlangen-Nürnberg (FAU), 91054 Erlangen, Germany; wei.xiang@fau.de

**Keywords:** alpha-Synuclein, melanocytes, melanosomes, pigmentation, pigment transport

## Abstract

Pigmentation is an important process in skin physiology and skin diseases and presumably also plays a role in Parkinson’s disease (PD). In PD, alpha-Synuclein (aSyn) has been shown to be involved in the pigmentation of neurons. The presynaptic protein is intensively investigated for its pathological role in PD, but its physiological function remains unknown. We hypothesized that aSyn is both involved in melanocytic differentiation and melanosome trafficking processes. We detected a strong expression of aSyn in human epidermal melanocytes (NHEMs) and observed its regulation in melanocytic differentiation via the microphthalmia-associated transcription factor (MITF), a central regulator of differentiation. Moreover, we investigated its role in pigmentation by performing siRNA experiments but found no effect on the total melanin content. We discovered a localization of aSyn to melanosomes, and further analysis of aSyn knockdown revealed an important role in melanocytic morphology and a reduction in melanosome release. Additionally, we found a reduction of transferred melanosomes in co-culture experiments of melanocytes and keratinocytes but no complete inhibition of melanosome transmission. In summary, this study highlights a novel physiological role of aSyn in melanocytic morphology and its so far unknown function in the pigment secretion in melanocytes.

## 1. Introduction

The understanding of the pigmentation process of the skin is still incomplete. Although mechanisms such as pigment synthesis itself are mostly known, further processes such as pigment transport and distribution of melanosomes to keratinocytes need deeper understanding. In skin diseases, from pigmentation disorders such as vitiligo to tumor entities such as malignant melanoma, pigmentation plays an important role [1,2,3,4]. To optimize therapies and to find new targets and therapeutic options, an enhanced knowledge of these molecular processes is needed.

Melanocytes as pigment-producing cells are in the focus of many researchers’ attention. They derive from unpigmented precursor cells called melanoblasts (MBrcs), which originate from embryonic neural crest cells and then mainly follow the dorso-lateral migration pathway [5,6,7,8,9,10,11,12]. One single differentiated melanocyte builds up the epidermal unit in the epidermis of the human skin and interacts with up to 36 keratinocytes [5,7,13,14]. Epidermal melanocytes are responsible for constitutive melanin production, which determines skin and hair color [5,7]. Among others, melanocyte-specific markers are tyrosinase (TYR), tyrosinase-related protein 1 (TYRP1), and DOPAchrome tautomerase (DCT or tyrosinase-related protein-2 (TRP2)). These enzymes are involved in pigment synthesis, while the microphthalmia-associated transcription factor (MITF) controls pigmentation gene transcription and melanocytic differentiation [5,15].

Melanin is synthesized and transported in intracellular organelles described as melanosomes [16,17], which are critical for protecting skin against UV radiation [18,19]. Mature melanosomes travel intracellularly along microtubule tracks and attach to the distal actin cytoskeleton via binding proteins including RAB27A (Ras-related protein RAB-27A). These transport complexes travel to the peripheral regions of the cell, the melanocytic dendrites, and accumulate at the distal tips [13,20,21]. The process of melanin secretion and uptake by the surrounding keratinocytes is still not well understood and discussed controversially [14,20,22,23]. By electron microscopy, it was suggested that the encapsulating membrane of melanosomes fuses with the plasma membrane of melanocytes, thereby leading to the secretion of melanin pigment (called melanocore or naked melanin). Next, the exocytosed melanocore is taken up by neighboring keratinocytes [20]. Other studies propose different transport processes, for example, a transport mechanism via tubules [13,20,24].

In our study, we aimed to elucidate the role of alpha-Synuclein (aSyn) in melanocytes and epidermal pigmentation processes. Most information known about the small 140-amino-acid protein stems from neuronal research, but its normal, physiological role in other cell types is still less investigated [25,26,27]. ASyn was shown to play an important role in neurodegenerative disorders and its aggregated forms are connected to the pathogenesis of Parkinson’s disease (PD) [28,29]. PD is characterized by a progressive loss of pigmented dopaminergic neurons in the substantia nigra [26] and was already linked to the development of malignant melanoma [30,31,32,33]. Additionally, aSyn has been shown to interact with lipid membranes of organelles, e.g., with synaptic vesicles, and is known to play an important role in synaptic transmission [26,34,35]. Pigmentation as well as vesicle transport are not exclusively restricted to neuronal cells and are even found in melanocytes. Therefore, we expect that aSyn is not only involved in the pathogenesis of PD but also a key molecule in melanocytic processes within the skin. We hypothesize that aSyn is involved in the pigmentation, differentiation, and/or melanosome trafficking of epidermal melanocytes.

In this study, we examined the influence and role of aSyn in melanocytic differentiation processes and morphology, as well as its effect on melanosome transport and transfer to keratinocytes. Our results provided new insights into the physiological role of aSyn in melanocytes and its impact on melanosome release.

## 2. Materials and Methods

### 2.1. Cell Lines and Culture Conditions

For the expression analysis, different human melanoma cell lines were used. For primary tumor studies, the cell lines *Mel Ei*, *Mel Ho*, *Mel Juso*, and *Mel Wei* were used. For human metastases of malignant melanoma *HMB2*, *Mel Im*, *Mel Ju*, and *SKMel28* cells were used. Human melanoma cell lines (*Mel Ei, Mel Ho, Mel Im, Mel Juso,* and *Mel Wei*) were described previously [36,37,38,39]. The human cell line *Mel Ju* was provided by Dr. Judith Johnson (LMU, Munich, Germany), *HMB2* cells were provided by Dr. Ian Hart (Queen Mary’s School of Medicine and Dentistry, London, UK), and *SKMel28* cells (HTB-72) were obtained from the American Type Culture Collection (ATCC, Virginia, VA, USA). *Mel Ju*, *HMB2*, and *SKMel28* cells were cultured as described [40]. All melanoma cell lines were split at a ratio of 1:3 to 1:5 on every 3rd day and incubated at 37 °C in a humidified atmosphere containing 8% CO_2_. *HaCaT* cells (keratinocytes, obtained from Cell Lines Service (CLS), Eppelheim, Germany) described by Boukamp et al. [41], and fibroblasts’ BJ1 cells, a gift from Dr. Ingo Thievessen (TRR225, FAU, Erlangen-Nürnberg, Germany), were used. HaCaTs were cultured and split like melanoma cell lines under a humidified atmosphere of 5% CO_2_ at 37 °C. Fibroblasts were cultured in DMEM high-glucose medium at 8% CO_2_ and split at a ratio of 1:3 two times a week. Human primary epidermal melanocytes derived from normal skin (NHEM, dark pigmented donor; PromoCell, Heidelberg, Germany) were cultivated in M2 melanocyte growth medium (M2 with Supplementary Mix containing growth factors and hormones (e.g., pituitary gland extracts with alpha-MSH), PromoCell, Heidelberg, Germany) under a humidified atmosphere of 5% CO_2_ at 37 °C. They were used between Passages 4 and 14 and split once per week at a ratio of 1:4. Melanocytes were de-differentiated into melanoblast-related cells (MBrcs) and cultured as previously described [42,43]. Co-culture experiments were based on previously described methods with modifications [44,45,46]. NHEMs and *HaCaTs* were seeded in a ratio of 1:5 and incubated as described. To separate the different cell types, differential trypsinization was performed. After separation, melanin content measurements, immunofluorescence staining, and further assays were performed as described below.

### 2.2. Tissue Samples

Human tissue samples were obtained from the tissue collection of the Institute of Pathology, University of Regensburg, Germany. Sampling and handling of patient material were carried out in accordance with the ethical principles of the Declaration of Helsinki. The use of human tissue material was approved by the local ethics committee of the University of Regensburg (Application Number: 09/11 and 03/151).

### 2.3. Transfection Experiments

Melanocytes were plated in a concentration of 1.0 × 10^5^ cells/well into 6-well plates. After three days of incubation, they were transfected with siPool (short interfering RNA pool) [47] targeting specifically aSyn (siSNCA) or MITF (siMITF, siTOOLs Biotech GmbH, Planegg/Martinsried, Germany). As previously described [48], the Lipofectamine RNAiMax reagent (Life Technologies GmbH, Darmstadt, Germany) was used for transfection according to the manufacturer’s instructions. Six hours after transfection, the culture medium was replaced and cells were incubated for 96 h before harvesting.

### 2.4. RNA Isolation, Reverse Transcription, and Quantitative RT-PCR

Total cellular RNA was isolated using the E.Z.N.A. MicroElute Total RNA Kit (Omega Bio-Tek, VWR Darmstadt, Germany) according to the manufacturer’s instructions. RNA concentration was measured by a NanoDrop spectrophotometer (Peqlab Biotechnologie GmbH, Erlangen, Germany). Complementary DNA was generated using reverse transcriptase (SuperScript II Reverse Transcriptase Kit (Life Technologies, Carlsbad, CA, USA)), with each reaction containing 500 ng of total RNA and 1 µL of enzyme as described previously [49]. Quantitative Real-Time PCR (qRT-PCR) was performed on the LightCycler 480 system (Roche, Mannheim, Germany) with an annealing temperature of 60 °C as previously described [50]. For analysis, specific primer sequences for each gene were used (Table 1). Beta-actin was used as a reference gene for normalization.

### 2.5. Transcriptome Analysis with cDNA Microarray

In this study, melanocytes and de-differentiated melanocytes were analyzed in three biological replicates (Supplementary Table S1). Sample processing was performed at an Affymetrix Service Provider and Core Facility, “KFB-Center of Excellence for Fluorescence Bioanalytics” (Regensburg, Germany; www.kfb-regensburg.de (accessed on 8 December 2021)) as described previously [36].

### 2.6. Protein Isolation and Western Blot Analysis

As described previously [48], cells were lysed in radioimmunoprecipitation assay (RIPA) buffer, and the protein concentration was determined using the Pierce BCA Protein Assay Kit (Thermo Fisher Scientific Inc., Rockford, IL, USA). For each sample, 30 µg of total protein lysate was separated on 12.75% SDS polyacrylamide gel electrophoresis. For analysis, the following primary antibodies were used: 1:5000 mouse anti-beta-actin (A5441, Sigma Aldrich, Munich, Germany), 1:2000 mouse anti-alpha-Synuclein (#610787, BD Transduction Laboratories, New Jersey, NJ, USA), 1:600 rabbit anti-TRP2 (ab74073, Abcam, Berlin, Germany), 1:500 mouse anti-MITF (sc-515925, Santa Cruz Biotech, Heidelberg, Germany), 1:500 mouse anti-TYR (sc-20035, Santa Cruz Biotech, Heidelberg, Germany), 1:1000 rabbit anti-RAB27A (#69295, Cell Signaling Technology, Frankfurt, Germany), 1:5000 rabbit anti-Cytochrome C (ab133504, Abcam, Berlin, Germany) and/or 1:1000 rabbit anti-GAPDH (#2118S, Cell Signaling Technology, Frankfurt, Germany). As secondary antibodies, horseradish peroxidase-coupled antibodies (1:2000, anti-rabbit HRP or anti-mouse HRP, Cell Signaling Technology, Frankfurt, Germany) were used, staining was performed by using the ECL Plus Western Blotting Detection Kit (GE Healthcare Life Science Europe GmbH, Freiburg, Germany), and luminescence was detected by the Intas ECL Chemocam LabImager.

### 2.7. Cell Fractionation

To separate cytosol and mitochondria, the ProteoExtract^®^ Cytosol/Mitochondria Fractionation Kit (QIA88, Calbiochem, Darmstadt, Germany) was used. Buffers were prepared as described in the manufacturer’s protocol. For cell fractionation, 15 × 10^5^ NHEMs were collected by centrifugation at 200× *g* for 4 min, prepared as described in the manufacturer’s protocol, and buffer amounts were adjusted to cell amounts. For homogenization, cells were passed 20 times through a syringe with a G27 nozzle and afterwards incubated on ice for further 5 min. The cell lysate was resolved in 200 µL of RIPA buffer and further described in the manuscript as “homogenate”. The cytosol and mitochondria fraction were separated as described in the protocol, and finally, equivalent amounts of the fractions were used for Western blot analysis as described above and separated on a 15% SDS polyacrylamide gel.

### 2.8. Melanin Content Determination

To quantify the melanin content, we followed previous publications, applying modifications [51,52,53,54,55]. For determination of the intracellular melanin content of si-treated NHEMs or *HaCaT* cells that were co-cultured with NHEMs and differentially trypsinized, 1.0 × 10^6^ cells were collected, centrifuged at 200× *g* for 4 min, and washed twice with PBS. For extracellular melanin quantification, melanosomes were isolated from the supernatant. To precipitate the melanin, pellets were dissolved in 1 N NaOH containing 10% dimethyl sulfoxide (DMSO, Sigma Aldrich, Munich, Germany) at 95 °C for 1 h. The absorbance was measured using a spectrophotometer at 415 nm, and the melanin content was calculated by interpolating the results with a standard curve of resolved synthetic melanin (M0418-100MG; Sigma Aldrich, Munich, Germany), generated by the absorbance of known concentrations.

### 2.9. Melanosome Isolation of Cell Culture Supernatant and Staining

The isolation of melanosomes from cell culture supernatant was performed by isolation via ultracentrifugation (UC) [56,57,58,59]. Therefore, cell culture media of three 6-well plated si-treated cells were collected after 96 h of transfection. The cell culture media was centrifuged at 1000× *g* and afterwards at 3000× *g* for 15 min at 4 °C to remove cell debris. Next, the isolation of the melanosomes was performed by centrifugation at 100,000× *g* for 1 h at 4 °C (Beckman Coulter Optima™ MAX-XP Ultracentrifuge, TLA55 rotor: k-factor 66, ref 366725). After washing the melanosomes with PBS they were frozen at −20 °C for further quantification of extracellular melanin or prepared for analysis via microscopy or fluorescence-activated-cell-sorting (FACS) in stained and unstained conditions. For staining isolated melanosomes, the ExoGlow™-Protein EV Labeling Kit (#EXOGP100A-1, Biozol, Eching, Germany) was used according to the manufacturer’s instructions.

### 2.10. Light Scattering

For characterization of released melanosomes by melanocytes, we performed light scattering analysis based on the publication of Wang et al. with modifications [60]. In this context, a higher absorbance correlates with a higher number of released melanosomes. Our analysis was performed using two different methodological implementations. A previous centrifugation of the supernatant at 3000× *g* for 10 min at 4 °C removed cell debris. For the first method, the absorbance of released melanosomes in the supernatant was directly measured by transferring 200 µL of supernatant onto a transparent plate in duplicates. Alternatively, the melanosomes in the supernatant were isolated by ultracentrifugation as described before, resolved in 400 µL PBS and measured in duplicates on a microplate reader. For background measurements, PBS or M2 melanocyte growth medium was used. Subsequently, the background value was subtracted from the sample values. All measurements were performed at 415 nm.

### 2.11. Immunofluorescence Analysis

For immunofluorescence assays, 15,000 NHEMs were seeded on cover slips in 12-well plates; for co-culture stainings, 60,000 *HaCaTs* and 15,000 NHEMs were used and incubated for two days. Fixation, permeabilization, and blocking were performed as described previously [61,62]. After blocking, cells were incubated with specific primary antibodies: mouse anti-alpha-Synuclein (1:200 #610787, BD Transduction Laboratories, New Jersey, NJ, USA), rabbit anti-TRP2 (1:500 ab74073, Abcam, Berlin, Germany), mouse anti-TYRP1 (1:100 sc-166857, Santa Cruz, Biotech, Heidelberg, Germany), rabbit anti-KRT14 (1:1000 HPA023040, Sigma Aldrich, Munich, Germany), and rabbit anti-Cytochrome C (1:1000 ab133504, Abcam, Berlin, Germany) at 4 °C overnight. After incubation with a secondary antibody (1:200 Alexa Fluor 488 anti-rabbit and/or anti-mouse IgG green fluorescence, Invitrogen, Darmstadt, Germany; 1:100 Cy3 anti-mouse IgG, Biozol, Eching, Germany; 1:1000 Alexa Fluor 555 anti-rabbit IgG red fluorescence, Invitrogen, Darmstadt, Germany), staining with 1:100 Rhodamine Phalloidin (PHDR1, 14 µM, tebu-bio, Offenbach, Germany) for 45 min and 1:1000 DAPI (0.1 mg∙mL^−1^, Merck KGaA, Darmstadt, Germany) for 30 min were performed. As mounting medium, Aqua-Poly/Mount (US Headquarters Polysciences, Warrington, PA, USA) was used. For analysis/control of the size of isolated and stained melanosomes, 10 µL of resolved melanosome solution was mounted with Aqua-Poly/Mount on a slide. Analysis was performed using an IX83 fluorescence microscope (Olympus, Hamburg, Germany) CellSens Dimension Software 1.12 (Olympus Cooperation) or the software Fiji based on ImageJ2 v2.1.0/1.53c (open source) for processing and analyzing scientific images. For quantification and analysis of the distribution of mitochondria via Cytochrome C and/or melanosomes by TYRP1, Phalloidin was used for the cellular restriction to measure the intensity of the respective antibodies. Additionally, background noise was deducted.

### 2.12. Fluorescence-Activated-Cell-Sorting (FACS)

To quantify the melanosomes isolated from cell culture media via flow cytometry, stained and unstained melanosomes were directly suspended in 400 µL of PBS after isolation or staining and analyzed using an LSRFortessa™ Cell Analyzer (BD Biosciences, San Jose, CA, USA). For cytometer settings, green fluorescent flow cytometry sub-micron beads (F13839, Invitrogen, Darmstadt, Germany) were used as size reference for small particles between 0.1 and 0.5 µm. Stained melanosomes were measured in the dark (excitation: 573 nm, emission: 588 nm, laser line 561 nm). For all measurements, the event counts for analysis remained the same, and all signals were detected (background gating took place after admission). Data were analyzed using FlowJo Software (V.10.7.1 LLC).

### 2.13. Statistical Analysis

GraphPad Prism 7 (GraphPad Software, Inc., San Diego, CA, USA) was used as a graphical and statistical tool. If not otherwise declared, at least three replicates were measured, and statistical analysis was performed using Student’s *t*-test. All results were given as the mean ± standard error of the mean (SEM). A value of *p* < 0.05 was considered statistically significant (ns: not significant, *: *p* < 0.05, **: *p* < 0.01, ***: *p* < 0.001, ****: *p* ≤ 0.0001).

### 2.14. Accession Numbers

Microarray data are available from NCBI Gene Expression Omnibus under Access Number GSE23034: Ohi et al. [63]; https://www.ncbi.nlm.nih.gov/geo/query/acc.cgi?acc=GSE23034 (accessed on 12 January 2022).

## 3. Results

### 3.1. Alpha-Synuclein Expression in Melanocytes

**Figure 1:**
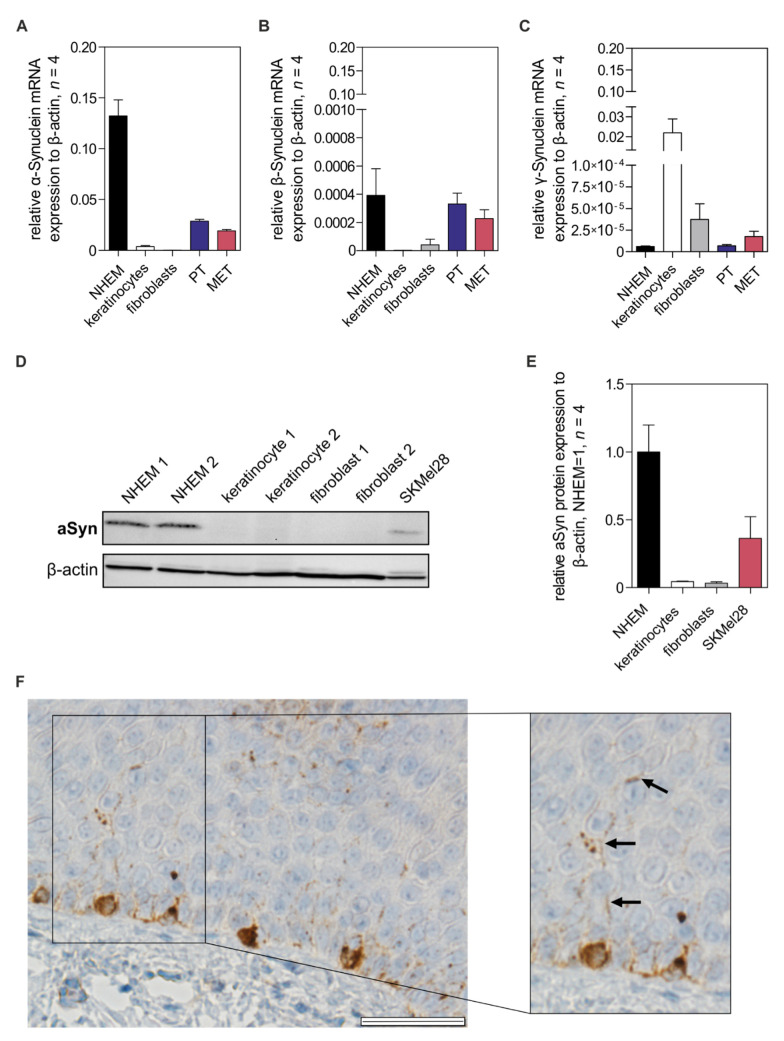
Expression analysis of the Synucleins, especially alpha-Synuclein (aSyn). (**A**) Relative mRNA expression of aSyn in different cell types of the skin (melanocytes = NHEMs, keratinocytes, fibroblasts, melanoma cell lines: primary tumor = PT, metastases = MET). For melanoma cells, four different cell lines were used: PT: *Mel Ho*, *Mel Juso*, *Mel Ei*, *Mel Wei*; MET: *HMB2*, *SKMel28*, *Mel Im*, *Mel Ju*. Cell lines were measured in triplicates. (**B**,**C**) Expression analysis of the Synuclein isoforms beta (**B**) and gamma (**C**) was performed by qRT-PCR as described in (**A**). (**D**,**E**) Protein analysis of aSyn by Western blot analysis in various cell lines (NHEMs, keratinocytes, fibroblasts, melanoma cells (*SKMel28*)) (*n* = 4). NHEMs were used for normalization (NHEM = 1). (**F**) Immunohistochemistry of normal human skin tissue samples demonstrated specific aSyn expression within melanocytic cell body and dendrites (marked by arrows, scale bar = 50 µm). For expression analysis, beta-actin was used for normalization. The box plots show the mean ± standard error of the mean (SEM).

To gain insight into the functional role of Synucleins in melanocytes, we first performed expression analysis by qRT-PCR on all Synuclein family members (alpha-, beta-, gamma-Syn), which exhibit a high sequence homology. Interestingly, alpha-Synuclein (aSyn) is strongly expressed in normal human epidermal melanocytes (NHEMs) and can be found in different melanoma cell lines (primary tumor = PT, metastases = MET) (Figure 1A). However, the Synuclein family members beta and gamma were only weakly expressed (Figure 1B,C). Therefore, we concentrated on aSyn and confirmed its expression on protein level in NHEMs (Figure 1D,E). We validated the detection of aSyn using the melanoma cell line *SKMel28*, where protein expression of aSyn has previously been shown [64]. To demonstrate aSyn expression in vivo, immunohistochemistry staining for aSyn in human skin tissue samples was performed. In melanocytes, strong expression was detected. Interestingly, aSyn staining was also found in the dendritic protrusions of melanocytes, illustrating the distribution and localization of the protein (Figure 1F). Our findings are supported by the staining published by the human protein atlas (https://www.proteinatlas.org/ENSG00000145335-SNCA/tissue/Skin#rnaseq, accessed on 5 January 2022; 09:27). For expression analysis, we found a specific aSyn expression in melanocytes compared to its family members.

### 3.2. Alpha-Synuclein Expression Is Regulated in Melanocytic Differentiation by MITF

**Figure 2:**
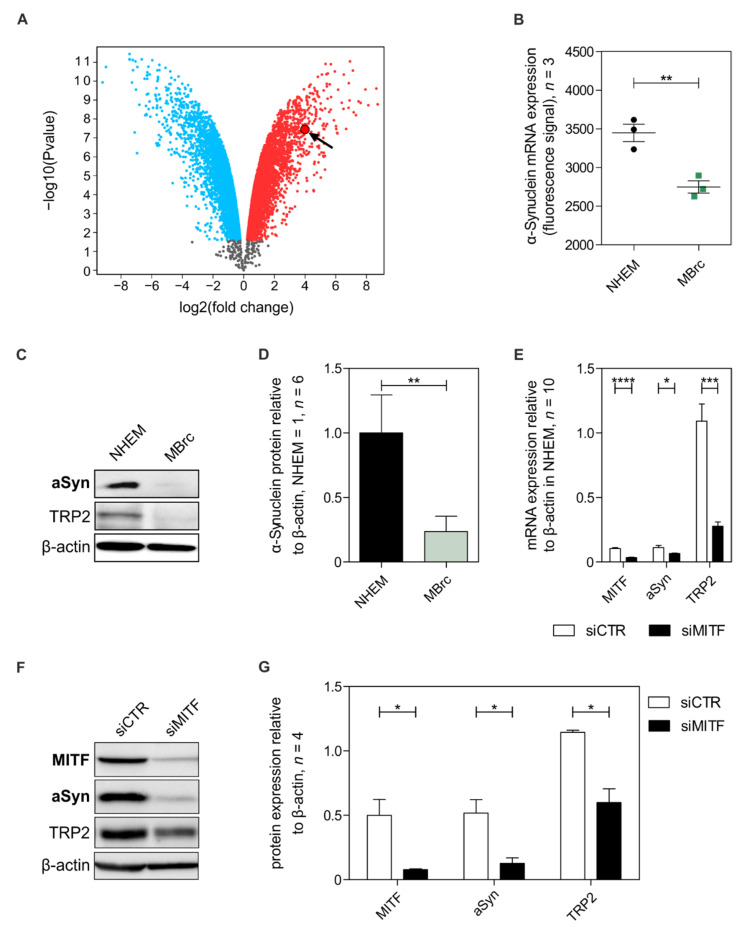
Regulation of aSyn by melanocytic differentiation and microphthalmia-associated transcription factor (MITF). (**A**) Volcano plot depicting aSyn expression by in silico analysis of a comparison of melanocytes and de-differentiated melanocytes (MBrcs) induced into pluripotent stem cells (IPs). Raw dataset of Ohi et al. (GSE23034) [63]. ASyn was marked with black arrow. (**B**) Evaluation of aSyn within transcriptome analysis data (*n* = 3) obtained by cDNA microarray (Supplementary Table S1) of melanocytes compared to MBrc. (**C**,**D**) Western blot analysis and densitometric evaluation of aSyn protein expression in melanocytes compared to MBrcs (*n* = 6). (**E**) Analysis of the effect of downregulated MITF on aSyn and tyrosinase-related protein-2 (TRP2) in NHEMs by qRT-PCR (*n* = 10). (**F**,**G**) Protein expression analysis of aSyn and TRP2 in siMITF-treated NHEMs (*n* = 4). Beta-actin was used as an internal control for normalization. The dot and box plots show the mean ± SEM. Paired *t*-test: *: *p* < 0.05, **: *p* < 0.01, ***: *p* < 0.001, ****: *p* ≤ 0.0001.

Based on differential expression patterns in melanoma cells and melanocytes, we speculated that aSyn expression correlates with differentiation. First, we performed in silico analysis on a dataset of melanocytes compared to de-differentiated melanocytes induced into pluripotent stem cells (IPs) (NCBI Gene Expression Omnibus, GSE23034) [63]. We compared these two groups via the web tool GEO2R and found a higher expression of aSyn in melanocytes compared to IPs (Figure 2A) and that aSyn is regulated depending on the differentiation status. To confirm our hypothesis, we cultivated de-differentiated melanoblast-related cells (MBrcs) generated by growth factor treatment of differentiated melanocytes [42,43]. Analysis of MBrcs on mRNA (Figure 2B, Supplementary Table S1) and protein level (Figure 2C,D) revealed a strong reduction of aSyn expression in MBrc compared to untreated NHEMs. As our data showed that aSyn is expressed in differentiated melanocytes, we analyzed the regulation of aSyn expression by the MITF, a central regulator and activator for melanocytic differentiation [5,65,66]. MITF downregulation in melanocytes using a siPool (siMITF) strongly reduced mRNA levels of aSyn (Figure 2E). Analyzing the protein level of aSyn after downregulation of MITF (Figure 2F,G), we confirmed that aSyn expression is induced in differentiation and regulated by the transcription factor MITF. Effects of siMITF were validated by controlling the reduction of TRP2, which is known to be regulated by the transcription factor MITF on mRNA and protein level (Figure 2E–G). So far, we demonstrated the regulation of aSyn in melanocytic differentiation and its regulation by MITF.

### 3.3. Influence of Alpha-Synuclein on Melanocytic Differentiation

**Figure 3:**
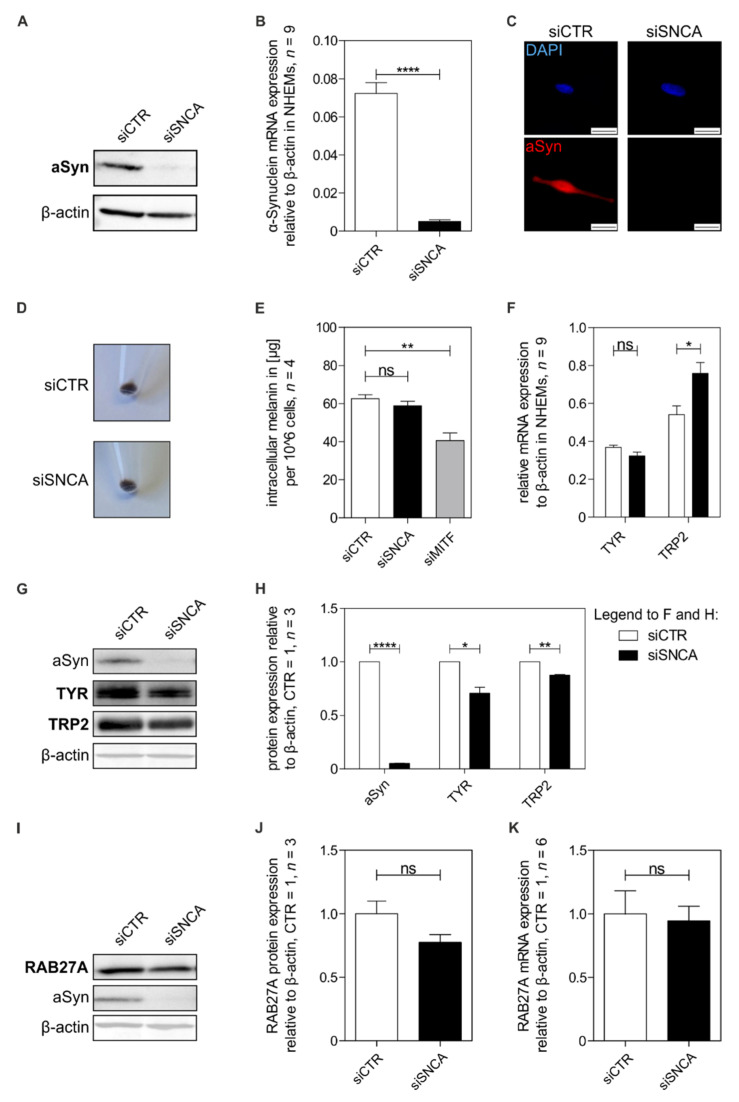
Analysis of aSyn in melanocytic differentiation. (**A**–**C**) Verification of aSyn knockdown in NHEMs on protein (**A**) and mRNA level (B) (*n* = 9) and by immunofluorescence staining (**C**). Stainings were performed using aSyn antibody (red) and DAPI (blue) on siCTR- and siSNCA-treated NHEMs. Scale bars represent 20 µm. (**D**) Depiction of the pellet color of NHEMs treated with siCTR versus siSNCA. (**E**) Measurement of the intracellular melanin content of 10^6 siCTR, siSNCA and/or siMITF-treated NHEMs (*n* = 4). (**F**) qRT-PCR measurements of the pigmentation genes TYR (tyrosinase) and TRP2 upon siSNCA treatment compared to control cells (*n* = 9). (**G**,**H**) Pigmentation analysis of protein level of siSNCA-knockdown cells compared to control cells with regard to pigmentation genes (TYR, TRP2; *n* = 3). (**I**,**J**) Protein analysis of the effect of aSyn knockdown on the transport protein RAB27A (*n* = 3). (**K**) qRT-PCR analysis of RAB27A in melanocytes treated with siSNCA or siCTR (*n* = 6). Beta-actin was used for normalization. The box plots show the mean ± SEM. Paired *t*-test: ns: not significant, *: *p* < 0.05, **: *p* < 0.01, ****: *p* ≤ 0.0001.

We next asked whether aSyn itself has an impact on melanocyte differentiation. We first established an siPool for downregulation of aSyn (siSNCA) gene expression and confirmed the knockdown on protein level (Figure 3A), mRNA level (Figure 3B), and further using immunofluorescence staining (Figure 3C). Next, we compared the pigmentation of siCTR cells with siSNCA cells and found no obvious differences in melanocyte pigmentation (Figure 3D). Analysis of the total intracellular melanin content of the cells also revealed no changes after siSNCA treatment, while treatment with siMITF reduced the melanin content significantly (Figure 3E). Further, only minor changes in the expression of pigmentation genes (TRP2, TYR) were observed (Figure 3F–H). These data suggest that aSyn is not involved in the regulation of melanin synthesis. In addition, we examined the effect of aSyn knockdown on RAB27A, involved in the transport of the mature melanosomes within melanocytes. We analyzed RAB27A protein (Figure 3I,J) and mRNA expression (Figure 3K) in aSyn knockdown (siSNCA) compared to control cells (siCTR) and found no significant differences in RAB27A expression levels. Taken together, we showed the regulation of aSyn in melanocytic differentiation but saw no influence on the pigmentation of melanocytes and no effects on the transport protein RAB27A by downregulation of aSyn.

### 3.4. Alpha-Synuclein and Mitochondria

**Figure 4:**
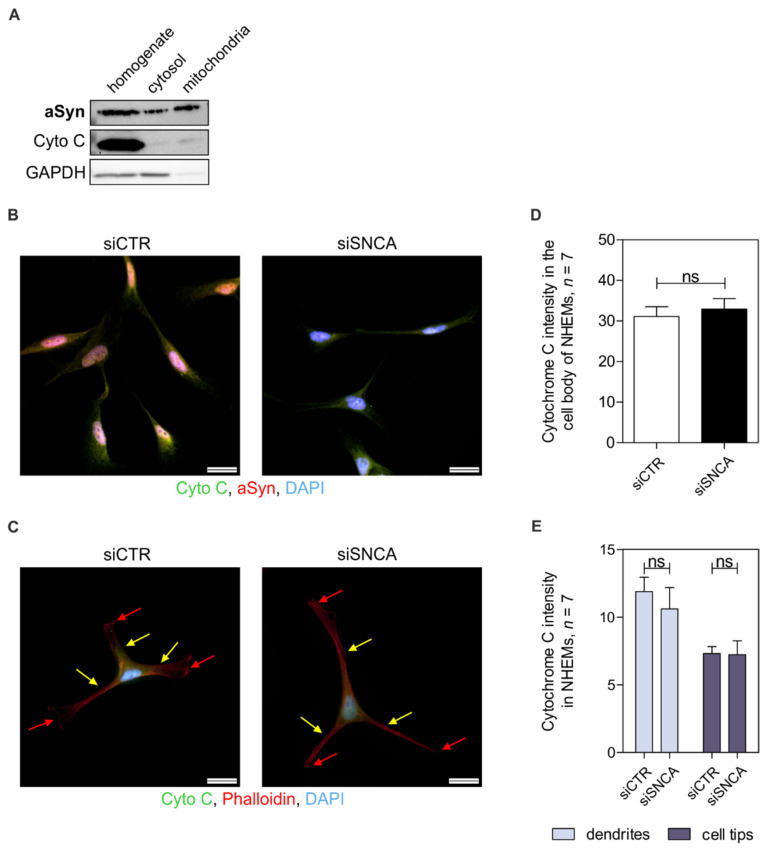
Analysis of the effect of aSyn on mitochondria. (**A**) Western blot analysis of the localization of monomeric aSyn via fractionated cell extraction. Cytochrome C (Cyto C) was used as a control for mitochondria, whereas GAPDH served as a control for the cytosolic fraction. (**B**) Immunofluorescence staining of Cytochrome C (green) and aSyn (red) in siCTR- and siSNCA-treated NHEMs. Scale bars represent 100 µm. (**C**) Staining of si-treated (siCTR vs. siSNCA) NHEMs with Cytochrome C (green) and Phalloidin (red); scale bars = 20 µm. For (**B**,**C**), DAPI (blue) was used for staining of the nucleus. Arrows highlight the melanocytic dendrites (yellow) and tips (red). (**D**) Quantification of the intracellular mitochondria dependent on the Cytochrome C staining depicted in (**C**) in NHEMs treated with siCTR or siSNCA (*n* = 7). (**E**) Detailed analysis of the mitochondria distribution in treated NHEMs within the melanocytic dendrites and the cellular tips (*n* = 7). For the quantifications depicted (**D**,**E**), the Phalloidin staining was used as cell boundary when measuring the intensity value of Cytochrome C. The box plots show the mean ± SEM. Paired *t*-test: ns: not significant.

It was already described that in PD aSyn has a specific functional role in mitochondrial homeostasis and distribution [67,68]. These highly dynamic organelles provide energy and regulate a variety of cellular processes in healthy conditions [69]. Therefore, we hypothesized that aSyn plays an important role in mitochondrial distribution and affects intracellular transport processes in melanocytes mediated by mitochondrial energy supply. We analyzed the cellular localization of aSyn by disruption of the melanocytic cell membrane and separation of the cellular components into cytosol and mitochondria, which were analyzed by Western blot. ASyn was observed in all fractions. GAPDH and Cytochrome C were used as controls for cytosol and mitochondria, respectively (Figure 4A). We performed immunofluorescence staining with antibodies against aSyn and Cytochrome C, respectively, in NHEMs treated with siCTR or siSNCA but revealed no obvious differences in the number or localization of mitochondria (Figure 4B). To quantify the staining of Cytochrome C in these cells, we performed co-stainings of Cytochrome C and Phalloidin, a marker for the actin cytoskeleton, to measure the intensity of the mitochondria in different parts of the cells (Figure 4C). We first analyzed the intensity of Cytochrome C staining within the whole cell body and found no difference between the siSNCA- and the siCTR-treated cells (Figure 4D). To exclude that a potential difference is not based on a reduced number of mitochondria but on the mitochondria transported to the cell dendrites (Figure 4C, yellow arrows) or tips (Figure 4C, red arrows), both parts of the cells were analyzed for the Cytochrome C intensity, but no significant differences were seen (Figure 4E). So far, our results suggest that aSyn knockdown does not influence melanocytic mitochondria.

### 3.5. Effect of Alpha-Synuclein on Intracellular Melanosome Distribution and Transport

**Figure 5:**
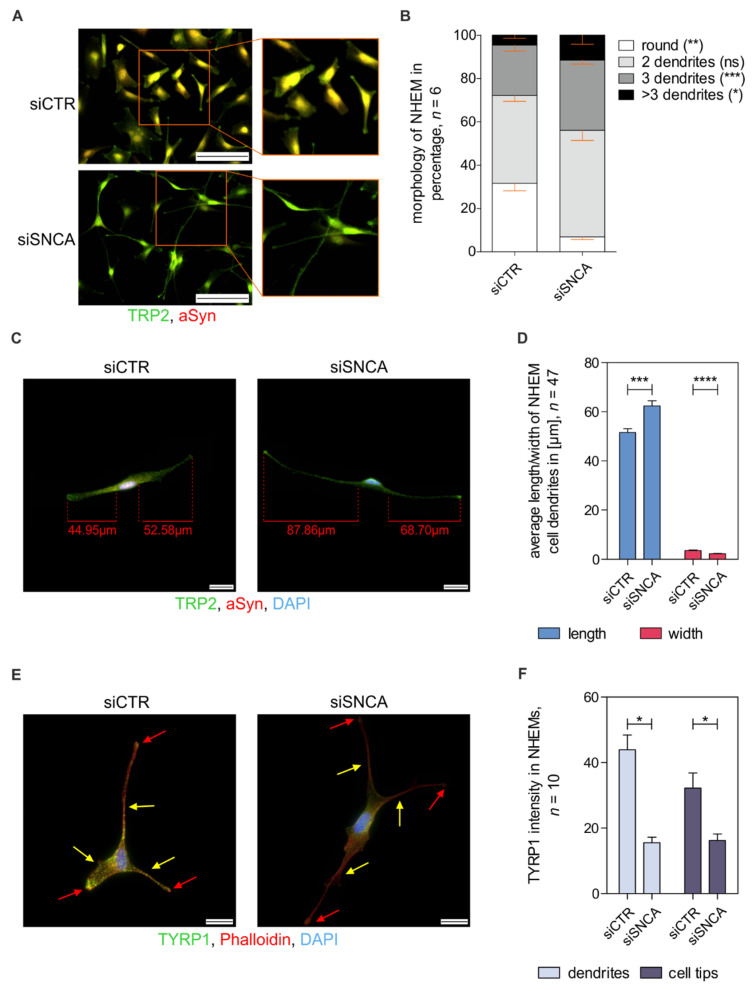
Examination of the effect of aSyn on melanosome distribution and transport. (**A**) Immunofluorescence staining of NHEMs treated with siCTR or siSNCA using anti-TRP2 (green) and anti-aSyn (red) antibodies showed morphological differences. Scale bars represent 100 µm. (**B**) Quantification of the morphological differences in melanocytic branching due to aSyn knockdown (*n* = 6). (**C**) Examples of si-treated (siCTR vs. siSNCA) NHEMs stained for TRP2 (green), aSyn (red), and DAPI (blue). Dendrites were measured in length and width (scale bars = 20 µm). (**D**) Quantitative analysis of the length and width of melanocytic dendrites with siCTR treatment or aSyn knockdown (*n* = 47). (**E**) Immunofluorescence staining of melanosomes for tyrosinase-related protein 1 (TYRP1, green), Phalloidin (red), and DAPI (blue) in siCTR- and siSNCA-treated NHEMs (scale bars = 20 µm). Arrows highlight the melanocytic dendrites (yellow) and tips (red). (**F**) Quantitative evaluation (*n* = 10) of transported melanosomes in the dendrites and cellular tips of si-treated NHEMs exemplary depicted in (**E**). Quantification of TYRP1 intensity was measured in dependency to the cell surface that was restricted by Phalloidin staining. The box plots depict the mean ± SEM. Paired *t*-test: ns: not significant, *: *p* < 0.05, **: *p* < 0.01, ***: *p* < 0.001, ****: *p* ≤ 0.0001.

Next, we concentrated on morphological changes of melanocytes after siSNCA treatment. Here, we analyzed the cells via immunofluorescence staining (Figure 5A) of TRP2 (green) and aSyn (red) and quantified the morphological changes (Figure 5B). SiSNCA treatment resulted in significantly enhanced branching of the melanocytes compared to siCTR-treated NHEMs (Figure 5B). Furthermore, we measured the length and width of the dendrites (Figure 5C) and demonstrated that siSNCA resulted not only in more intensely branched cells but also in significantly longer and thinner dendrites (Figure 5D). To investigate the intracellular effects of the morphological changes and the effect on the intracellular transport of melanosomes from the nucleus to the cell tips along the dendrites, we stained the siSNCA-treated cells for TYRP1 (green), a melanosomal marker within melanocytes, and Phalloidin for the actin cytoskeleton (red, Figure 5E). For quantification of the immunofluorescence signal of TYRP1 in the dendrites (Figure 5E, yellow arrows) and in the dendritic tips (Figure 5E, red arrows) of the cells, Phalloidin was used as a cell boundary. Interestingly, we observed a significant reduction in the transported melanosomes (green) in both dendrites and dendritic tips of siSNCA-treated melanocytes (Figure 5F). Obviously, aSyn does not affect pigmentation but influences the morphology of melanocytes as well as the transport of melanosomes.

### 3.6. Role of Alpha-Synuclein on Melanosome Release and Transfer

**Figure 6:**
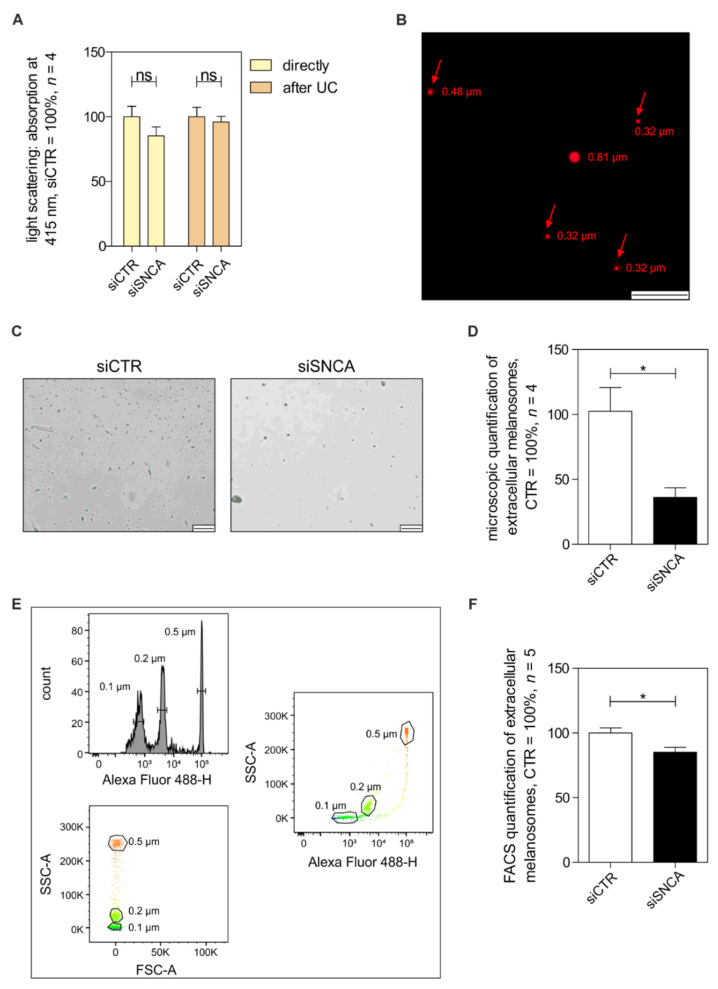
Analysis of the role of aSyn on melanosome release. (**A**) Absorption measurement of the melanosomes released by siCTR or siSNCA-treated NHEMs via light scattering analysis (*n* = 4). The absorption was measured twice: directly in the supernatant of the treated cells and after melanosome enrichment by ultracentrifugation (UC) of the supernatant. (**B**) Isolated and stained melanosomes as quality and size control for the isolation of extracellular vesicles by UC. Red arrows mark isolated vesicles corresponding to the expected size of melanosomes. Scale bar represents 5 µm. (**C**) Microscopic bright-field images of vesicles isolated from the supernatant of siCTR- and siSNCA-treated NHEMs (scale bars = 20 µm). (**D**) Quantification of extracellular melanosomes by microscopic bright-field imaging (*n* = 4). (**E**) Fluorescent-activated-cell-sorting (FACS) of green fluorescent beads in different sizes for establishment of subsequent quantifications of released melanosomes. (**F**) FACS measurement of released melanosomes isolated by UC of siSNCA-treated NHEMS dependent on the measured event counts (*n* = 5). The box plots show the mean ± SEM. Paired *t*-test: ns: not significant, *: *p* < 0.05.

After we investigated the intracellular effects of aSyn on the distribution and transport of melanosomes, we wanted to gain insight into further effects of aSyn on melanosome release and transfer. Therefore, we quantified the melanosomes in the cell culture supernatant released from siSNCA-treated cells. First, we performed light scattering experiments on the supernatant of the treated melanocytes and revealed a reduction in the absorbance of the medium of siSNCA-treated melanocytes, suggesting a reduced number of released melanosomes (Figure 6A). Next, we performed melanosome isolation from the supernatant by ultracentrifugation (UC) and confirmed that the isolated vesicles were melanosomes using an extracellular vesicle staining kit and fluorescence microscopy to analyze the vesicle size (Figure 6B, arrows). Furthermore, microscopic analyses also suggested a reduction in the number of released vesicles (Figure 6C, D). To quantify the isolated melanosomes and compare the number of melanosomes released from siSNCA-treated NHEMs, we additionally performed flow cytometry measurements. For cytometer settings of forward (FSC) and sideward scatter (SSC) for small particles, we used fluorescent beads between 0.1 and 0.5 µm as a size reference (Figure 6E). Quantification of extracellular melanosomes revealed a significant reduction of melanosome release after aSyn silencing for event-dependent measurements (Figure 6F).

**Figure 7:**
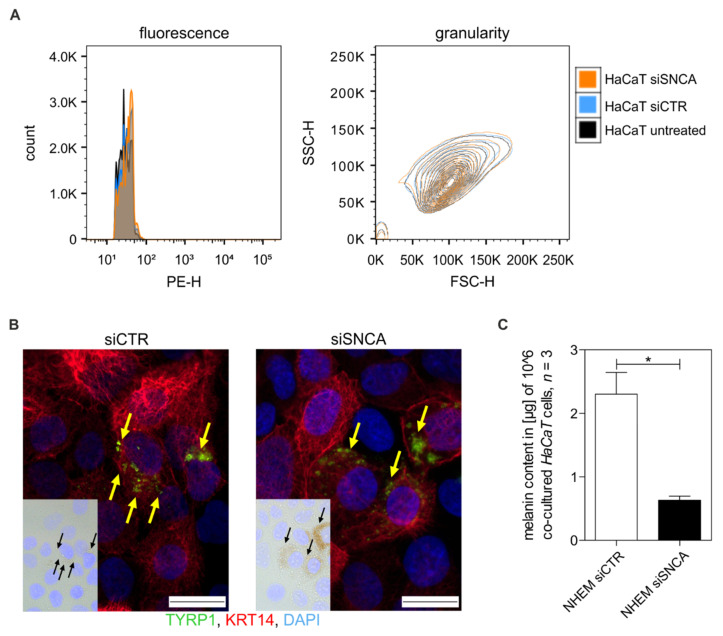
Effect of aSyn on melanosome transfer to keratinocytes. (**A**) FACS analysis of the uptake of fluorescent melanosomes and changes in the granularity of keratinocytes treated with isolated and stained melanosomes. (**B**) Bright-field image and immunofluorescence staining of co-cultured keratinocytes and melanocytes treated with siCTR or siSNCA using TYRP1 (green) and anti-keratin14 (KRT14, red) antibodies. DAPI (blue) was used to stain the nucleus. Black and yellow arrows show melanosomes absorbed by keratinocytes, scale bars represent 20 µm. (**C**) Analysis of the intracellular melanin content of keratinocytes that were co-cultured with si-treated NHEMs and differentially trypsinized (*n* = 3). The box plots depict the mean ± SEM. Paired *t*-test: *: *p* < 0.05.

After we demonstrated a reduction of melanosomes released by siSNCA-treated NHEMs compared to si-control treated cells, we investigated the transfer of melanosomes from NHEMs to keratinocytes in detail. Therefore, we isolated melanosomes derived from siSNCA-treated or siCTR-treated NHEMs, stained them with an extracellular vesicle staining kit, added them to adherent keratinocytes, and incubated the cells between four hours to four days. We evaluated the potential uptake of the stained melanosomes by harvesting the keratinocytes and analyzing their fluorescence signal and granularity. Here, we observed no differences within the fluorescence or granularity of the keratinocytes (Figure 7A). These results suggest that keratinocytes need the cell-cell contact of melanocytes for melanosome transfer. Consequently, we performed co-culture experiments of keratinocytes (red) and melanocytes (green) treated with siCTR versus siSNCA and demonstrated by bright-field and immunofluorescent imaging that, in general, melanosomes were transferred to keratinocytes in both siRNA treatments (Figure 7B, black and yellow arrows). Interestingly, quantification of the melanin content of co-cultured HaCaTs revealed a reduced uptake of melanosomes by co-cultured melanocytes treated with siSNCA (Figure 7C).

Based on the morphological changes, we demonstrated that aSyn downregulation resulted in a reduction of melanosome release and transfer to keratinocytes and that the knockdown of aSyn led to a reduced transport of mature melanosomes to the dendritic tips. In summary, we showed that aSyn plays a crucial role in melanosome secretion in melanocytes.

## 4. Discussion

Several studies have observed a central role of aSyn in the pathogenesis of neurodegenerative disorders such as PD [35]. PD, which is described as a loss of pigmented dopaminergic neurons in the substantia nigra by an accumulation of aSyn [26], was also associated with a variety of dermatological disorders [32]. This also includes malignant melanoma [30,31], a skin cancer caused by a malignant transformation of melanocytes [3,70,71] and mal-pigmentation [3]. However, less is known about the impact of aSyn in melanocytes, which are responsible for skin pigmentation.

In this study, we aimed to analyze the so far unknown physiological role of aSyn in epidermal melanocytes, pigmentation, and melanin transport via melanosomes. We observed a strong expression of aSyn in NHEMs, which is consistent with the findings of other studies [30,31,63,64]. Investigations into the role of aSyn in melanocytic differentiation showed a higher expression level in differentiated melanocytes compared to MBrcs. This was also supported by our re-analysis of a dataset from Ohi et al., who de-differentiated melanocytes into induced pluripotent stem cells and analyzed the cells via cDNA microarray [63]. ASyn expression is induced during melanocytic differentiation by the MITF, a central regulator of melanocyte development, that can promote differentiation-activated functions and regulates genes implicated in pigmentation (e.g., TYR, TYRP1, and TRP2) [31,72,73]. Interestingly, we found no information about MITF being involved in neuronal differentiation (https://www.proteinatlas.org/ENSG00000187098-MITF, accessed on 2 May 2022; 15:24); hence, a cell-type-specific regulation can be demonstrated in melanocytes.

Melanocytes are responsible for skin pigmentation [7]; however, analysis of the role of aSyn in pigmentation demonstrated no effects on the intracellular melanin content. These data suggest that aSyn, despite being a downstream target of MITF, is not involved in the regulation of pigmentation. These results are supported by the observation that the homozygous aSyn knockout mice do not show obvious changes in the intensity of skin pigmentation [74,75]. In PD, characterized by changes in aSyn expression or protein form, a premature death of pigmented dopaminergic neurons in the substantia nigra results in depigmentation [76]. These strong cellular effects are not observed in melanocytes. Pan et al. described that aSyn was associated with pigmentation in melanoma using aSyn overexpression experiments and UV treatment, leading to a reduction in melanin synthesis [53]. They hypothesized that aSyn may interact with TYR, inhibiting UV-induced TYR activation [53]. In contrast to their hypothesis, we saw no direct impact of aSyn on pigment synthesis in aSyn knockdown experiments in melanocytes; however, we did not analyze UV treatment on melanocytes.

ASyn was described to be also localized and bound to mitochondria in neurons, and overexpression and loss were proposed to result in mitochondrial dysfunction [35]. It was described that aSyn deletion affects lipid composition in the brain, and mitochondrial abnormalities were observed by comparable amounts of mitochondria [77,78]. Our analysis supports the localization of aSyn to mitochondria, but we found no changes in the amount or distribution of mitochondria within the melanocytes or defined cellular compartments after knockdown of aSyn. Here, obviously, aSyn has cell-type-specific functions or is differently regulated in melanocytes compared to neurons.

Interestingly, after aSyn knockdown, changes in melanocytic morphology were found. We observed a higher amount of dendritic spreading, as well as longer and thinner dendrites within siSNCA-knockdown cells. Regarding Phalloidin staining, the actin cytoskeleton seemed to be intact and was not changed in shape. Studies on aSyn in the neuronal system and PD also showed effects of altered aSyn expression levels resulting in morphological changes in neurite length and branching [79,80]. ASyn overexpression was also described to inhibit tubulin polymerization, which is important for neurite elongation and is implicated in actin cytoskeleton formation and neurite branching [79,80].

Investigating the transport and release of melanin-bearing vesicles in melanocytes, we demonstrated a reduced secretion and transfer of melanosomes. These new findings were also supported by further results, revealing a reduction in melanosomes within the dendrites and cellular tips, quantified by TYRP1 staining. This is in agreement with studies in PD, describing that aSyn not only results in a loss of dopaminergic neurons [81] but also impairs the axonal transport of synaptic vesicles [79]. Mature melanosomes are transported from the perinuclear region to the periphery of melanocytes via the transport complex of RAB27A–melanophilin–myosin Va on the actin filament [21,82,83]. We, therefore, concentrated on the direct impact of aSyn on RAB27A but found no change after siSNCA treatment. Studies in neuronal systems demonstrated interaction of aSyn and SNARE-complex (soluble N-ethylmaleimide-sensitive-factor attachment receptor) molecules mediating synaptic vesicle fusion and neurotransmitter release [84,85]. Here, aSyn appears to play an important role as a regulator for complex formation and SNARE-dependent vesicle fusion by promoting vesicle docking [84,85,86]. Additionally, Scott et al. identified SNARE proteins that are associated with melanocytic cell membranes [87,88] and showed that melanosomes contain the SNARE accessory protein called alpha-SNAP [87,88]. Therefore, we suggest a possible interaction of aSyn and SNARE-complex proteins in melanosome release.

So far, the mechanism of the melanosome transfer from melanocytes to the surrounding keratinocytes remains controversial. However, several studies show that more than one transfer mechanism (e.g., exocytosis–endocytosis, cytophagocytosis, shedding vesicle model, membrane fusion) is plausible [23,24,89]. This is also supported by our results, as a reduction in melanosome transfer of melanocytes by aSyn knockdown was measured, but no complete inhibition of transmission was seen. This suggests that aSyn knockdown could inhibit one or multiple transmission mechanisms, but not all.

## 5. Conclusions

Taken together, these findings shed light on the physiological role of aSyn in melanocytes. Our research indicates that aSyn has a major impact on cell morphology in melanocytes and is important for pigment transfer. Therefore, it could be of interest to analyze aSyn in future studies regarding pigmentation disorders to elucidate whether aSyn could be a potential biomarker.

## Figures and Tables

**Table 1:** Oligonucleotide sequences and qRT-PCR conditions.

Primer	Forward Primer 5′-3′	Reverse Primer 5′-3′	Product Size in bp	T_m_ in °C
ACTB	CTACGTCGCCCTGGACTTCGAGC	GATGGAGCCGCCGATCCACACGG	384	88
MITF	TCTACCGTCTCTCACTGGATTGG	GCTTTACCTGCTGCCGTTGG	141	83
RAB27A	TGATGGAGCGAACTGCTTTTC	CCCTACACCAGAGTCTCCCAA	296	78
SNCA	GCAGAAGCAGCAGGAAAGAC	TTCCTGTGGGGCTCCTTCTT	232	86
SNCB	CGTGTTCATGAAGGGCCTGT	GTGAGGCCTGTTCCTTGGTT	187	88
SNCG	GACCTCAGTGGCCGAGAAGAC	CTCTTCAGGTCATCCACGCT	261	89
TRP2	TGGAGTGGTCCCTACATCCTA	TCACTGGTGGTTTCTTCCG	165	85
TYR	CTCAAAGCAGCATGCACAAT	GCCCAGATCTTTGGATGAAA	265	82

T_m_ = Melting temperature.

## Data Availability

External data sources used in this study are cited in the article. The extracted data is available in Supplementary Materials.

## Supplementary Materials

The following supporting information can be downloaded at: https://www.mdpi.com/article/10.3390/cells11132087/s1, Table S1: cDNA microarray dataset of NHEMs and de-differentiated NHEMs (=MBrcs).
